# Influence of Colostrum and Vitamins A, D_3_, and E on Early Intestinal Colonization of Neonatal Holstein Calves Infected with *Mycobacterium avium* subsp. *paratuberculosis*

**DOI:** 10.3390/vetsci6040093

**Published:** 2019-11-20

**Authors:** Judith Stabel, Lucas Krueger, Caitlin Jenvey, Taylor Wherry, Jesse Hostetter, Donald Beitz

**Affiliations:** 1 US Department of Agriculture-Agricultural Research Service (USDA-ARS), National Animal Disease Center, Ames, IA 50010, USA; C.Jenvey@latrobe.edu.au (C.J.); Taylor.Wherry@usda.gov (T.W.); 2 Department of Animal Science, Iowa State University, Ames, IA 50011, USA; Lucas.Krueger@agriking.com (L.K.); dcbeitz@iastate.edu (D.B.); 3 Department of Veterinary Pathology, Iowa State University, Ames, IA 50011, USA; jesseh@iastate.edu

**Keywords:** dairy calf, microbiome, colostrum, vitamins, *Mycobacterium avium* subsp. *paratuberculosis*

## Abstract

Exposure of neonates to *Mycobacterium avium* subsp. *paratuberculosis* (MAP) via infected dams is the primary mode of transmission of Johne’s disease. Little is known about the impacts of feeding colostrum and supplemental vitamins on the gut microbiome in calves exposed to MAP. In the present study, calves were assigned at birth to one of six treatment groups: (1) Colostrum deprived (CD), no vitamins; (2) colostrum replacer (CR), no vitamins; (3) CR, vitamin A; (4) CR, vitamin D_3_; (5) CR, vitamin E; (6) CR, vitamins A, D_3_, E, with five calves per treatment in a 14-day study. All calves were orally inoculated with MAP on days 1 and 3 of the study. Differences due to vitamin supplementation were not significant but treatment groups CR-A, CR-E, and CR-ADE had higher numbers of MAP-positive tissues overall. Shannon diversity indices demonstrated regional differences in microbial communities, primarily Proteobacteria, Bacteroidetes, and Firmicutes, between the ileum, cecum, and spiral colon of all calves. CD calves exhibited increased richness compared with CR calves in the cecum and spiral colon and harbored increased Proteobacteria and decreased Bacteroidetes in the mucosa compared with the lumen for all three tissues. Overall, supplementation with vitamins did not appear to influence gut microbiome or impact MAP infection. Feeding of colostrum influenced gut microbiome and resulted in fewer incidences of dysbiosis.

## 1. Introduction

Johne’s disease (paratuberculosis), a chronic enteritis caused by *Mycobacterium avium* subsp. *paratuberculosis* (MAP), can result in profuse diarrhea, malabsorption of nutrients, and severe weight loss in end-stage disease [1]. Since MAP infection is spread primarily through contaminated feces and/or milk and colostrum, efforts to mitigate disease transmission have focused on preventing infection in the neonate with clean maternity pens and feeding pasteurized colostrum and waste milk [2]. In addition to MAP infection, high incidences of morbidity in neonates are attributed to infection with enteric pathogens such as *E. coli*, *Salmonella*, coronavirus, rotavirus, *Cryptosporidia*, and *Campylobacter* [3]. Neonatal calves are immunosuppressed by maternal influences during late gestation but are also influenced by postnatal nutrition via lactocrine mechanisms that favor Th2 signaling [4,5]. Th1 and Th2 immune responses are more widely recognized in neonatal physiology for interaction with pathogens that cause acute, rather than chronic infections. The chronicity of Johne’s disease from a long latent period of asymptomatic infection to advanced clinical disease correlates with the development of Th2 immunity [1]. Thus, a dominant Th2 immunity observed for neonatal calves may favor uptake of MAP by the gut. Th2-mediated immunity is also precipitated by feeding of colostrum to neonates. Colostrum is a nutritive staple of commercial calf husbandry that is known to provide immunoglobulin G_1_ (IgG_1_) and other bioactive immune factors for passive transfer of immunity [6]. Colostrum biases toward Th2 signaling has been associated with increased disease resistance and survival [5,7]. Colostrum contains a broad range of components that are engaged in promoting intestinal health, including immunoglobulins, lactoferrin, cytokines, and maternal cells such as lymphocytes, macrophages, and neutrophils [5]. Failure of passive transfer may result in increased risk of morbidity [8] caused by major enteric pathogens such as rotavirus, coronavirus, and bovine viral diarrhea virus, as well as intracellular (*Salmonella*, *Clostridia*) and extracellular (*E. coli*, *Pseudomonas*) bacterial pathogens [9]. The period of time during which passively transferred immunity wanes, but endogenous immunity remains immature, is a critical window for pathogen opportunism. Neonatal susceptibility to disease during this window has been further linked to the dietary adequacy of vitamins A, D, and E [10,11]. Vitamins A, D, and E, and the many bioactive compounds in colostrum are known to affect epithelial maturation, membrane integrity, gene expression, and immune regulation that may collectively impact microbial colonization in the intestine [6,12,13,14,15].

The present study was conducted to evaluate the effects of supplementation with colostrum and vitamins A, D, and E on MAP colonization of the gut of the neonatal calf. Concomitant characterization of the effects of these nutrients on the commensal microbial community in lumen contents and mucosa of the intestine and cecum of MAP-infected calves was also performed. The study was designed to test the hypothesis that colostrum, vitamin supplementation, or both nutritional treatments may shift the microbial community and influence protection against MAP infection in young calves.

## 2. Materials and Methods

### 2.1. Study Design

**Figure 1:**
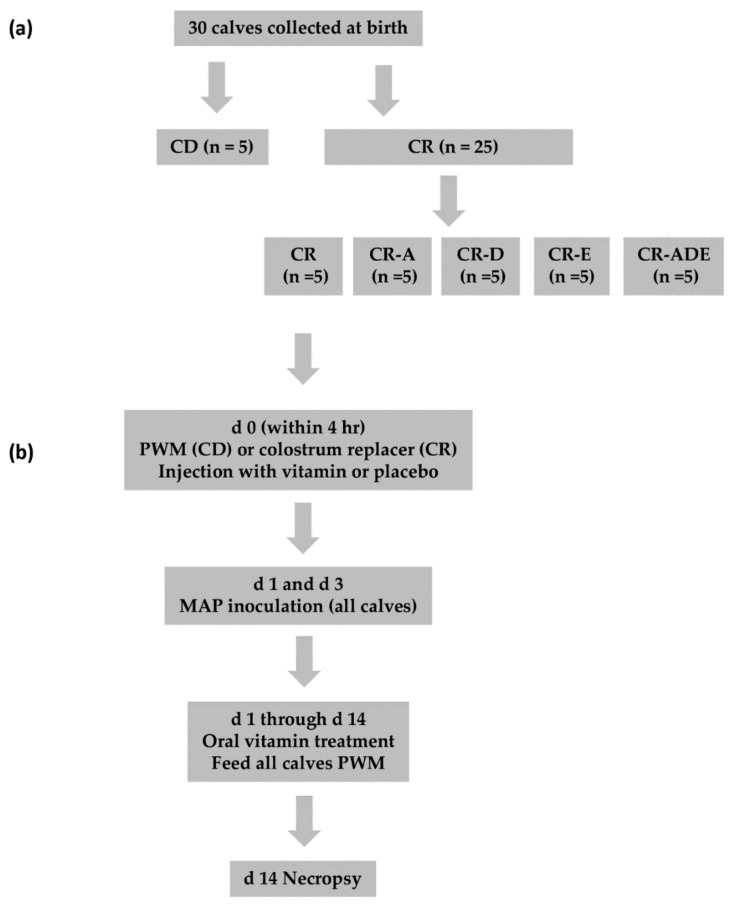
Experimental design (**a**), with five calves per treatment group. CD: Colostrum deprived; CR: Colostrum-replete; CR-A: Supplemented with vitamin A; CR-D: Supplemented with vitamin D_3_; CR-E: Supplemented with vitamin E; CR-ADE: Supplemented with vitamins A, D_3_, and E. Outline of experimental procedures (**b**). PWM: Pasteurized whole milk.

Thirty male Holstein calves were obtained at birth from two local dairy farms that were tested negative for Johne’s disease and randomly assigned to one of six treatment groups: (1) Colostrum deprived (CD), no vitamins; (2) colostrum replacer (CR), no vitamins; (3) colostrum replacer, vitamin A (CR-A); (4) colostrum replacer, vitamin D_3_ (CR-D); (5) colostrum replacer, vitamin E (CR-E); (6) colostrum replacer, vitamins A, D_3_, and E (CR-ADE), with five calves per treatment in a 14-day study (Figure 1) [16]. All calves received a first feeding of colostrum replacer or pasteurized whole milk (PWM) within 4 h of birth; the CD calves were fed 1.9 L of PWM (Iowa State University Dairy Farm, Ames, IA, USA) as a control, whereas calves in the remaining five treatment groups received 375 g of fractionated colostrum replacer (Milk Products, Chilton, WI, USA) reconstituted in 1.9 L water. The colostrum replacer contained 150 g bovine globulin protein concentrated from colostral whey and was devoid of detectable fat-soluble vitamins A, D_3_, and E. Retinol and α-tocopherol were quantified in PWM samples and averaged 31.2 ± 2.4 and 4996 ± 461 ng/mL, respectively, with no vitamin D_3_ detected. Calves received their respective vitamin treatments at the time of first feeding and throughout the study. Calves in vitamin treatment groups were administered 3 mL injections subcutaneously at birth to deliver 150,000 IU of retinyl palmitate (CR-A), 150,000 IU of cholecalciferol (CR-D), and 1500 IU of d-α-tocopherol (CR-E) (Stuart Products, Inc., Bedford, TX, USA). This was done to assure adequate administration of vitamins shortly after birth as some calves might have difficulty suckling. Calves in the CD and CR control groups were injected with 3 mL of placebo carrier solution at birth (proprietary formulation, Stuart Products, Inc., Bedford, TX, USA). Thereafter (days 1–14 of study), calves assigned to vitamin treatment groups were orally administered 25,000, 5000, and 500 IU of the respective compounds daily in their dietary milk. The CR-ADE calves received an injection or oral solution containing vitamins A, D_3_, and E in the concentrations described above.

### 2.2. Animal Care

All animal procedures performed were approved by the Animal Care and Use Committee, National Animal Disease Center, Ames, IA (Protocol#2675), and animals were cared for according to guidelines of the Federal Animal Welfare Act of 1966 and the Federation of Animal Science Societies, “Guide for the Care and Use of Agricultural Animals in Research and Teaching”. Calves were housed indoors in individual pens elevated from a concrete floor. Calves were placed on a twice-daily feeding schedule with 12 h intervals. At each feeding, calves were fed 2.7 L of PWM that was transported daily from the Iowa State University Dairy Farm, Ames, IA, USA. Calves received 1 mL of oral vitamin solution via dietary milk at each evening feeding, according to treatment group. All calves were bottle fed, but refusals of greater than 0.1 L were administered to the calf via esophageal tube. All calves were inoculated twice via dietary milk with 10^8^ cfu per dose of *Mycobacterium avium* subsp. *paratuberculosis* (MAP), strain 167 (clinical isolate, NADC, Ames, IA, USA) in PWM, as previously described [16] during the morning feedings of days 1 and 3. The study was designed to evaluate effects of colostrum and vitamins on early MAP infection so there were no uninfected calves in this study. Treatment groups were compared across calves within a MAP-infection model (Figure 1).

### 2.3. Fecal Culture and DNA Extraction

Fecal samples (2 gm) were obtained at day 7 and 14 and then processed by a centrifugation and double-decontamination procedure [17]. Decontaminated samples (200 µL) were dispensed onto Herrold’s Egg Yolk medium (HEYM) and incubated at 39 °C for 12 weeks. Tissues obtained at necropsy were homogenized in 0.9% hexadecylpyridinium chloride solution with a gentleMACS^TM^ Octo Dissociator, using M tubes (Miltenyi Biotec, San Diego, CA, USA). Following overnight incubation, homogenates were pelleted by centrifugation at 900× g for 30 min and resuspended in an antibiotic cocktail containing 100 µg/mL nalidixic acid, 100 µg/mL vancomycin, and 50 µg/mL amphotericin B (Sigma-Aldrich, St. Louis, MO, USA). Samples were incubated overnight and then inoculated onto four slants of HEYM. Slants were incubated at 39 °C for at least 12 weeks and colony counts of viable MAP were recorded and averaged for the four slants. Confirmation of MAP colonies was performed by picking colonies off the HEYM slants, boiling in ultra-pure water^TM^ distilled water (Life Technologies) to release the DNA, followed by IS900 PCR as described below. DNA from fecal samples was extracted by using the Ambion^®^ Mag Max^TM^ total nucleic acid isolation kit (Life Technologies, Grand Island, NY, USA) according to the manufacturer’s recommendations in conjunction with a Mag Max^TM^ Express processor (AM1840 v2, Applied Biosystems, Life Technologies, Beverly, MA, USA).

### 2.4. Tissue Culture, DNA Extraction, and Histopathology

Calves were euthanized after a 12 h fast on day 14 ± 1 for collection of 22 tissues, which included ileum, ileo-cecal valve, regions of jejunum, duodenum, cecum, spiral colon, transverse colon, descending colon, and lymph nodes of each region, as well as liver and spleen. Lumen contents of ileum, cecum, and spiral colon were collected from respective tissues into 15 mL conical tubes and placed on ice, followed by storage at −80 °C. Tissues were rinsed with 0.15 M PBS and cut into multiple cross-sections. Cross-sections of ileum, cecum, and spiral colon were opened to expose the mucosa, and a sterile glass slide was used to scrape the mucosal surface. Mucosal scrapings were collected into 1.5 mL microfuge tubes and stored at −80 °C until analyses could be performed. Additional sections of tissues were stored at −80 °C for culture of MAP. Cross-sections of tissues were fixed in formalin, embedded in paraffin, cut at 4–6 µm, and stained by using the Ziehl–Neelsen technique for visualizing acid-fast bacteria. Tissues were assigned scores on a scale of 0 to 5 based upon the presence of granulomatous lesions and acid-fast bacteria, where 0 represented granulomas/AF bacteria were not detected; 1, rare granulomas/AF bacteria were present in a section; 2, a granuloma with AF bacteria was typically present in each field; up to a score of 5 in which granulomas and AF bacteria were diffusely present and replaced most of the architecture as described within Jenvey et al. [18]. DNA was extracted from tissues by using modified procedures of the UltraClean^®^ tissue & cells DNA isolation kit (Mo Bio Laboratories, Inc., Carlsbad, CA, USA). Briefly, 100 mg of tissue was placed into a 2 mL screw cap vial, which contained 0.5 mL silica beads (0.1 mm diameter, BioSpec Products, Bartlesville, OK, USA), 700 µL of TD1 buffer (Mo Bio) and 20 µL proteinase K (Qiagen, Valencia, CA, USA). Samples were vortexed for 10 min, incubated at 60 °C for 1 h, and then UltraClean^®^ kit (company, city, country) procedures were performed according to the manufacturer’s instructions. PCR was performed for the detection of the IS900 target gene in feces and tissues by method previously described [19], with the exception that TaqMan Environmental Master Mix (Applied Biosystems, Foster City, CA, USA) was used in the reaction. The primer sequences used were 5′-CCGCTAATTGAGAGAT GCGATTGG-3′ for the forward primer, and 5′-AATCAA CTCCAGCAGCGCGGCCTCG-3′ for the reverse primer. The fluorescent probe sequence was 5′-FAM-TCCACGC CCGCCCAGACAGG-TAMRA-3′.

### 2.5. 16S Sequencing Protocols

Total DNA was extracted from lumen contents and mucosal scrapings of ileum, cecum, and spiral colon for amplification of the 16S ribosomal gene for phylogenetic sequencing. Samples (about 300 mg) were homogenized by using a gentleMacs^TM^ Octo Dissociator with M tubes (Miltenyi Biotec) containing 2.0 mL buffer (Qiagen). Homogenates were transferred to a 12 × 75 mm tube and mixed by rotating overnight with 50 µL proteinase K per 250 mg sample (Qiagen) at 56 °C. DNA was extracted by using the PowerMag^TM^ Microbiome RNA/DNA Isolation Kit^®^ (Mo Bio Laboratories, Inc.) according to manufacturer’s instructions. A Biomek^®^ FXP Laboratory Automation Workstation with DTX 880 mulitmode detector (Beckman Coulter, Inc., Indianapolis, IN, USA) was utilized for magnetic separation of DNA-binding beads and wash and elution procedures. Extracted samples were quantified using a Nanodrop 8000 spectrophotometer (Thermo Scientific, Waltham, MA, USA) and DNA concentration in each sample was brought to 500 ng/µL in water. Reaction conditions, generic construction of primers, and the index sequences used in the present study have been described [20], but primer sequences specific to the V1 and V3 regions (8F: 5′-AGAGTTTGATCCTGGCTCAG and 518R: 5′-ATTACCGCGGCTGCTGG) are described [21], and pad adaptor sequences were modified according to manufacturer’s specifications for the MiSeq Gene and Small Genome Sequencer (Illumina, San Diego, CA, USA). Sequencing was performed on the Illumina MiSeq platform using the 2 × 250 bp protocol and employing the Schloss MiSeq Wetlab SOP (https://github.com/SchlossLab/MiSeq_WetLab_SOP). The paired sequencing reads were quality filtered and the pairs were joined into contigs using the Poisson binomial filtering algorithm and the overlap joiner implemented in moira [22]. The moira contig constructor was set to report the best qscore, and filtering by moira was parameterized to treat ambiguous bases as sequencing errors. A custom Perl script was used to reformat the moira sequence and name files into the contigs FASTA file and the group file needed for analysis using mothur v. 1.39.5 [23]. The amplicon contigs were analyzed using the Schloss MiSeq SOP [20] except where differences were noted. Amplicons were screened to remove all sequences containing ambiguous bases and to contain sequences 364–500 bp long. Duplicate sequences were counted and collapsed into unique representative sequences. These unique sequences were aligned to the V1–V3 region of the Silva v. 128 non-redundant alignment database. After alignment, the amplicons were screened to remove sequences that did not properly align, and to remove any sequences that contained any homopolymeric nucleotide runs of eight or more bases. Sequences were again counted and then collapsed into unique representative sequences. Pre-clustering was performed, allowing a maximum of 10 total base pair differences (pre.cluster parameter “diff = 5”). Chimeric amplicons were removed with USEARCH and UCHIME (using the Silva v. 128 alignment database). Sequences were taxonomically classified using trainset16_022016 as a reference and specifying a cutoff of “80”. Singleton sequences that occurred in only one sample group were removed from the analysis [24]. This workflow resulted in the identification of 7361 unique sequences that represented 2,565,359 total amplicons. The operational transcriptional units (OTUs) were determined by calculating the distances between the unique sequences at a 97% level, (cutoff = 0.03), and then clustering the amplicons at this same level.

### 2.6. Microbial Community Data Analysis

OTUs were assigned to consensus taxonomy and were indexed according to sample. Cecum, spiral colon, and ileum lumen contents and mucosa were normalized by subsampling to 2263 sequences per sample. All microbial community data analysis was performed using R version 3.6.1 (R Foundation for Statistical Computing, Vienna, Austria). The package ‘phyloseq’, (version 1.28.0) [25] was used to import and analyze the OTU and taxonomy data. The alpha and beta diversity, relative and differential abundance of microbial populations was assessed within each tissue and sample. A Shannon diversity index was calculated for each sample, within each tissue, using the package ‘vegan’, version 2.4-5 [26]. Box plots of the Shannon diversity index for each sample were created using ‘ggplot2′, version 2.2.1 [27], and statistical comparisons were assessed using a Kruskal–Wallis ANOVA and a post-hoc Dunn Test with Bonferroni correction, using the package ‘dunn.test’, version 1.3.5 [28]. *p*-Values ≤ 0.05 were considered statistically significant. Non-metric multidimensional scaling (NMDS) was performed for each tissue and compared for each sample. NMDS ordination using a Bray–Curtis dissimilarity method was calculated using the package ‘funfuns’, version 0.1.0 [29] and the ‘NMDS_ellipse’ function, which returns a list containing three items; (1) input metadata with NMDS coordinates, (2) a data.frame containing information about the standard error ellipses for each group, and (3) the ordination object created by the ‘vegan’ function metaMDS. The NMDS plots with standard error ellipses were created using ‘ggplot2′. Statistical comparisons were assessed using the package ‘vegan’ and the ‘adonis’, ‘betadisper’ and ‘permutest’ functions. *p*-Values ≤ 0.05 were considered statistically significant. The differential and relative abundance of microbial populations was calculated for each tissue and compared for each sample using the package ‘phyloseq’. The relative abundance of Phyla was compared for each tissue and sample, using the ‘tax_glom’ function. Phyla that contributed more than 2% of the relative abundance of each sample was included in the analysis using the ‘transform_sample_counts’, ‘psmelt’, ‘filter’ and ‘arrange’ functions. Stacked bar plots of Phyla abundance were plotted against sample, for each tissue, using the package ‘ggplot2′. For differential abundance analysis. phyloseq microbiota data were converted to DESeq2 datasets and analyzed using the Wald test with a parametric fit to test for significance (*p* ≤ 0.05). All plots were generated using the package ‘ggplot2′. To lessen the impacts of rare OTUs, the raw OTU counts were agglomerated at the genus level.

## 3. Results

### 3.1. MAP Infection in Neonatal Calves

All 30 calves were healthy and thriving upon initiation of the study. One CR-D calf demonstrated protracted scouring during the 14-day study so that animal was supplanted with a new calf and not included in the experimental analyses. Fecal shedding of MAP at day 7 was detected in 18 of 28 calves by culture, with no sample obtained from two of the 30 calves. There were no determinant differences among treatment groups due to variability in degree of shedding and the number of calves that were shedding. However, calves in the CR-ADE group averaged 153 cfu/g feces compared to a range of 521 to 1972 cfu/g for other treatment groups (Table 2). By day 14 of the study, fecal shedding of MAP had declined substantially for all treatment groups with an average number of only one calf per group shedding MAP compared to an average of three calves per treatment on day 7 (Table 1). Performance of IS900 PCR on fecal samples resulted in the detection of 10 additional calves shedding MAP on day 7 although treatment effects were still inapparent.

Tissues obtained from calves at necropsy were assessed for the presence of MAP by culture and PCR during the 14-day infection period. Viable MAP was recovered from 42 of the 660 tissues collected at necropsy for the 30 calves. Nineteen of the 30 calves had positive tissues and these calves were distributed amongst all treatment groups (2–5 calves per treatment group). The average number of tissues positive for viable MAP was higher for calves in the CR-A, CR-E, and CR-ADE groups than calves in the CD, CR, and CR-D treatment groups (9.3 versus 3.3 tissues, respectively; *p* < 0.05). Of the 22 tissues taken from each calf, primary sites for MAP colonization were the distal jejunum, ileum, and ileo-cecal valve. Within these three target tissues there was a consistent presence of MAP recovered by culture across treatment groups except for the CD calves (Table 2). In addition, the degree of colonization by location did not differ in these three main culture-positive tissues, averaging 2.27 ± 0.89, 2.56 ± 0.87, and 2.41 ± 0.82 cfu/g in distal jejunum, ileum, and ileo-cecal valve, respectively. The low level of colonization corresponded with a lack of detectable lesions within the selected tissues, distal jejunum, ileum, and ileo-cecal valve, regardless of treatment group. Interestingly, CD calves had only three culture positive tissues overall and these were the transcending and spiral colon from one calf and the mesenteric lymph node from another calf.

Further determination of the presence of MAP in tissues was done by PCR quantification of the IS900 target gene, with no differences noted between tissue types among treatment groups. The distribution of positive tissues was more expansive than observed for culture, with all 22 tissues recording at least one positive result. Primary positive tissues paralleled that for tissue culture and included distal jejunum, ileum, ileo-cecal valve, cecum, and descending and spiral colons across all treatment groups. In agreement with culture, the number of PCR-positive tissues was once again higher for CR-E and CR-ADE calves.

### 3.2. Microbial Diversity

**Figure 2:**
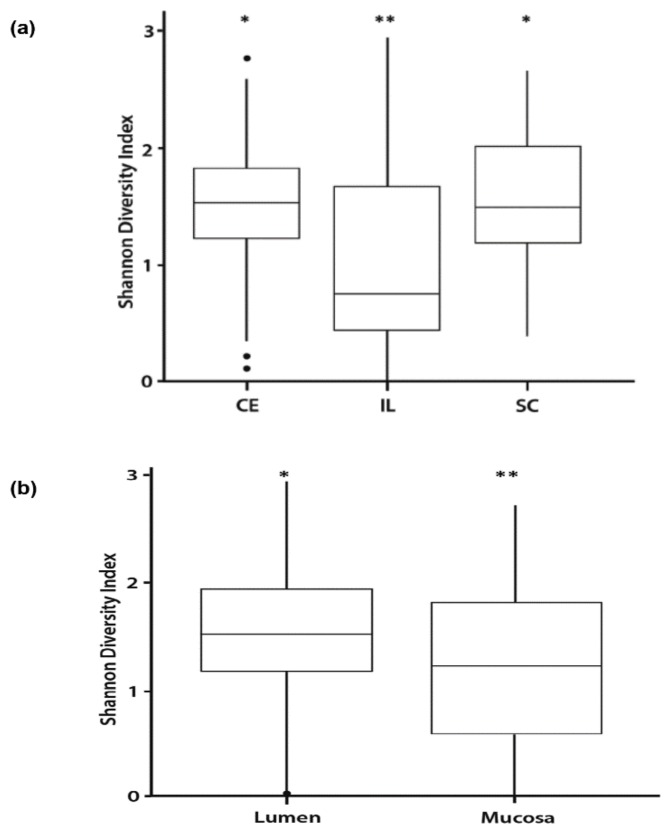
DNA was extracted from the lumen and mucosa of three intestinal tissues: Cecum (CE), ileum (IL), and spiral colon (SC) of calves and 16S sequencing was performed on the Illumina MiSeq Sequencer employing the Schloss MiSeq Wetlab protocol. All microbial community data analysis was performed using R version 3.6.1. Shannon diversity indices of microbial communities were calculated for each sample, within each tissue, using the package ‘vegan’, version 2.4-5. Shannon diversity indices for cecum, ileum, and spiral colon collapsed across treatments are presented, with greater values indicating increasing diversity within the tissue (**a**). Different superscripts are statistically different, *,** *p* < 0.001. Shannon diversity indices of microbial communities by either lumen contents or mucosa of all three major intestinal tissues; cecum, ileum, and spiral colon of calves are presented (**b**). Different superscripts are statistically different, *,** *p* < 0.02.

A Shannon diversity index was used to measure richness, evenness, and α-diversity in the lumen contents and mucosal scrapings of the cecum, ileum, and spiral colon. Regional effects in communities demonstrated significant differences (*p* < 0.001) between ileum, and the cecum and spiral colon (Figure 2a). Further delineation of communities between the lumen contents and mucosal scrapings showed that there was greater richness (*p* < 0.02) within the lumen contents of the three tissues collectively (Figure 2b).

**Figure 3:**
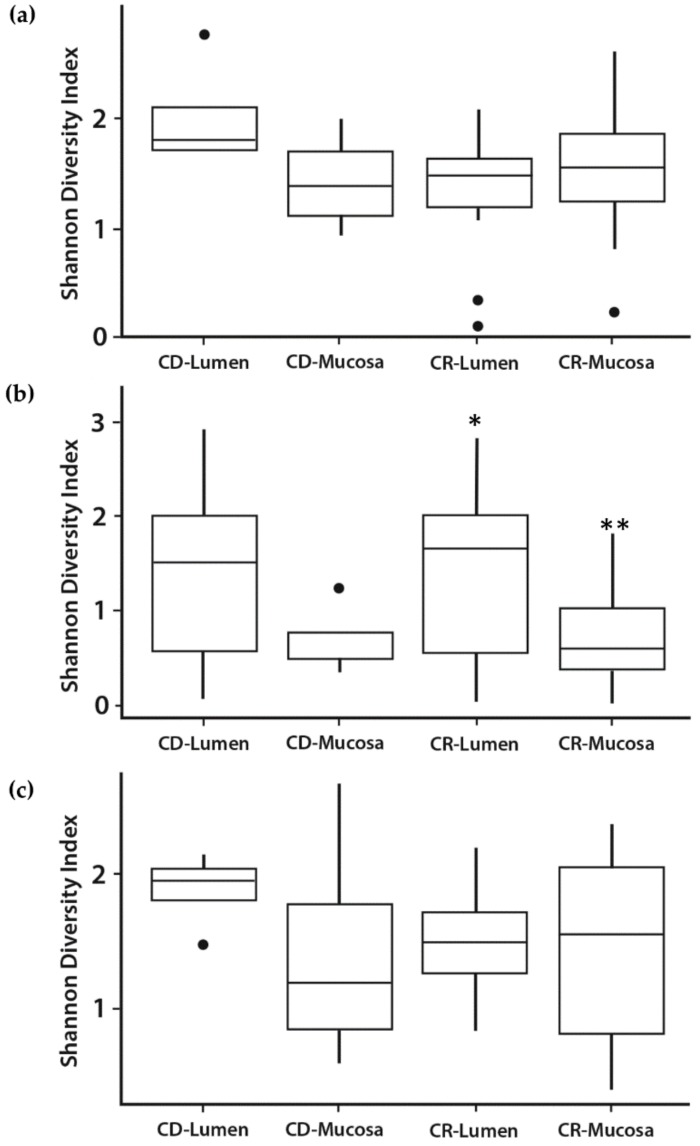
Shannon diversity indices of microbial communities were calculated for each sample, within each tissue, using the package ‘vegan’, version 2.4-5. Shannon diversity indices in the lumen and mucosa of three major intestinal tissues, cecum (**a**), ileum (**b**), and spiral colon (**c**) in calves not fed colostrum, CD = colostrum deprived, and calves fed colostrum, CR = colostrum replacer. Closed circles, •, represent outliers. Different superscripts are statistically different: Ileum, CR-Lumen versus CR-Mucosa, *,** *p* < 0.03.

The stratification between lumen contents and mucosa was more pronounced for the ileum with significant differences (*p* < 0.03) observed for CR calves (Figure 3b). In contrast, differences were observed only for CD calves in cecum and spiral colon tissues (Figure 3a,c). No differences were noted due to vitamin supplementation.

**Figure 4:**
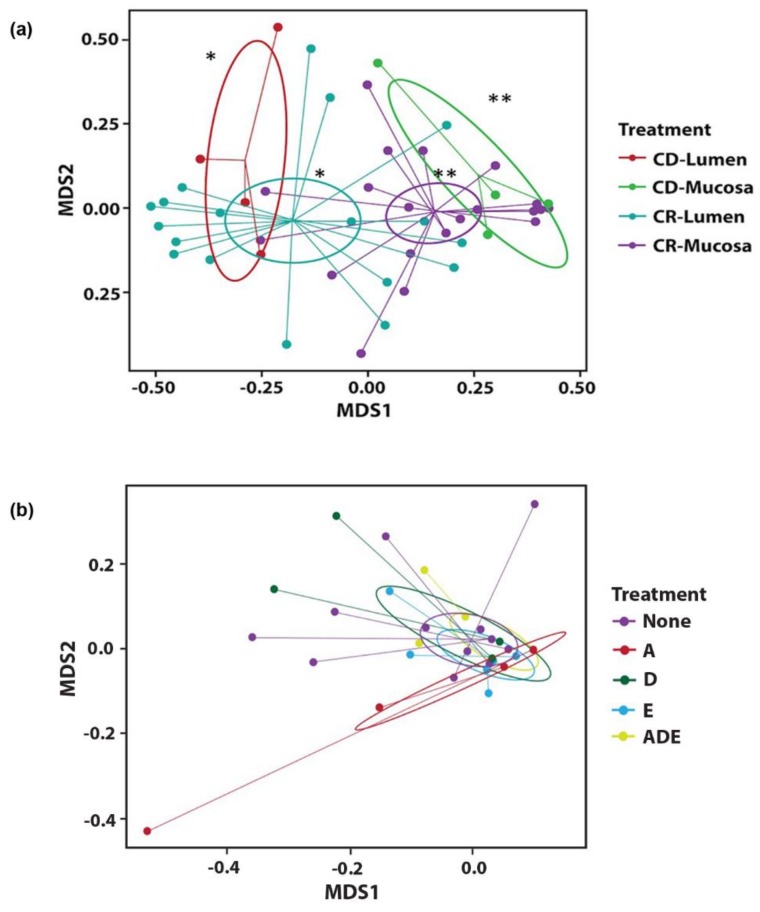
Non-metric multidimensional scaling (NMDS) was performed for each tissue and compared for each sample. NMDS ordination using a Bray–Curtis dissimilarity method was calculated to determine differences in microbial community due to nutritional treatment. The NMDS plots with standard error ellipses were created using ‘ggplot2′. Beta-diversity indices measured within lumen contents and mucosa of the spiral colon in calves not fed colostrum, CD = colostrum deprived, and calves fed colostrum, CR = colostrum replacer (**a**). Different superscripts are statistically different, CD-Lumen versus CD-Mucosa, *,** *p* < 0.03; CR-Lumen versus CR-Mucosa, *,** *p* < 0.0002. Beta-diversity indices for overall effects of supplementation with vitamins A, D, E, or a combined triad of ADE, measured within the ileum in calves (**b**). Samples were collapsed across lumen contents and mucosa within each treatment group.

Beta-diversity analyses of microbial communities demonstrated significant effects (*p* < 0.001) due to sampling location (lumen versus mucosa) for all calves across all three tissues. Differences were more apparent for CR calves (*p* < 0.0002) than CD calves (*p* < 0.04) overall, with results for spiral colon shown as an example (Figure 4a). There were no effects noted across the three tissues in either lumen contents or mucosa due to supplementation with vitamins, with results for the ileum shown as an example (Figure 4b).

**Figure 5:**
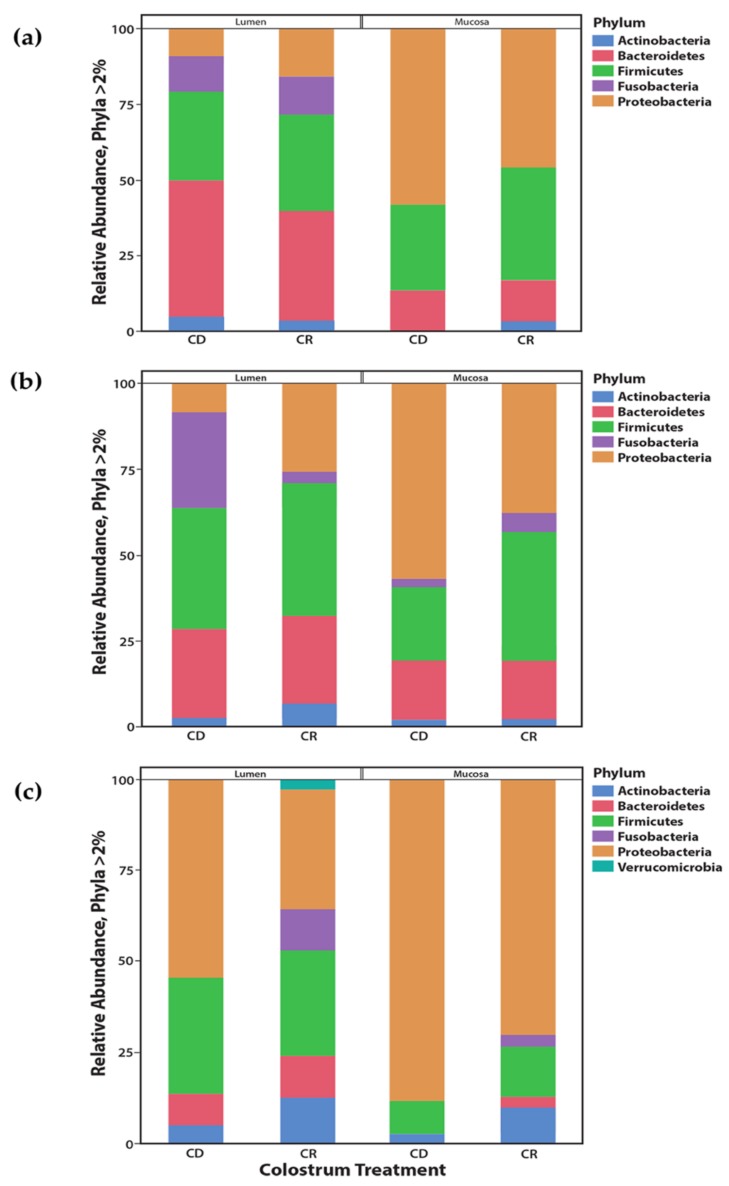
Relative abundance of microbial populations was calculated for each tissue and compared for each sample using the package ‘phyloseq’ and ‘tax_glom’ functions, respectively. Phyla that contributed more than 2% of the relative abundance of each sample were included in the analysis. Stacked bar plots of Phyla abundance were plotted against sample, for each tissue, using the package ‘ggplot2′. Mean relative abundance in the lumen contents and mucosa of the spiral colon (**a**), cecum (**b**), and ileum (**c**) in calves not fed colostrum, CD = colostrum deprived, and calves fed colostrum, CR = colostrum replacer. Mean phylum relative abundance differences between lumen and mucosa across all three tissues were apparent for Phylum Proteobacteria, *p* < 0.0001 and Phylum Bacteroidetes, *p* < 0.02.

Phylum assignments were given to each OTU based upon consensus taxonomy generated in mothur and phylum relative abundance was compared among lumen and mucosal samples from CD and CR calves within the spiral colon, cecum, and ileum (Figure 5a–c). Partitioning the phyla analyses into either lumen or mucosal locations resulted in overall differences (*p* < 0.0001). Proteobacteria, Bacteroidetes, and Firmicutes comprised 25.8%, 25.8%, and 33.0% of lumen content communities and 25.8%, 16.4%, and 29.2% of mucosal communities, respectively. An increased relative abundance of Proteobacteria (*p* < 0.0001) in the mucosa of the spiral colon was concomitantly associated with a decreased abundance of Bacteroidetes (*p* < 0.0001; Figure 5a). Within the cecum and ileum, Proteobacteria were higher (*p* < 0.02) and Firmicutes were lower (*p* < 0.02) in the mucosa compared to the lumen contents (Figure 5b,c).

**Figure 6:**
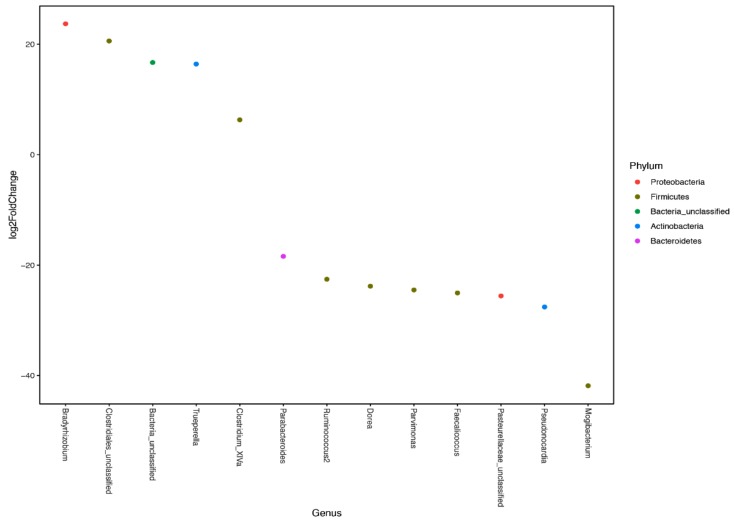
For differential abundance analysis. Phyloseq microbiota data were converted to DESeq2 datasets and analyzed using the Wald test with a parametric fit to test for significance. All plots were generated using the package ‘ggplot2′. To lessen the impacts of rare operational transcriptional units (OTU), the raw OTU counts were agglomerated at the genus level. Log2 fold change in differential abundance at the genus level within phyla was compared between genera present in the ileal mucosa for CR and CD calves, respectively. Color of dots represents the main phyla analyzed and position of dots represent increase or decrease in representation of specific genera due to colostrum feeding. Genera shown are statistically significant at *p* < 0.05.

Feeding colostrum resulted in differences in phyla in the mucosa and lumen of all three tissues but differences were defined within each tissue (Figure 5a–c). Overall, CD calves had higher (*p* < 0.0001) abundance of Proteobacteria, Bacteroidetes, Firmicutes, and Fusobacteria, depending upon tissue. Additionally, colostrum status of calves resulted in a shift to a significantly (*p* < 0.0001) higher abundance of Proteobacteria in the mucosa when compared to the lumen contents for CD calves for all three tissues sampled, however, this observation was only seen in the spiral colon (*p* < 0.001) and ileum (*p* < 0.0001) for CR calves (Figure 5a–c). In contrast, there was a greater abundance of Firmicutes (*p* < 0.03) in the mucosa for CR calves compared to other phyla. Relative abundance of Actinobacteria was low, averaging 7.2% and 4.5% for lumen contents and mucosa, respectively, across tissues but trended higher for CR calves. On a genus level, greater relative abundance was ascribed to populations of *Escherichia/Shigella*, *Bacteroides*, *Pseudomonas*, *Faecalibacterium*, and *Lachnospiraceae*, regardless of tissue or colostrum status. Cecum and spiral colon aligned more closely with one another, with Escherichia/Shigella averaging about 35% relative abundance to the total microbiome compared to 75% for the ileum. Interestingly, CD calves differed from CR calves with a 5% relative abundance of *Fusobacterium* in the cecum and 14% relative abundance of *Campylobacter* in the ileum. As the major target tissue for MAP colonization, the identity of the predominant genera within the ileum was of more interest. Due to the young age of calves at the time of sample collection, microbial colonization levels were low, resulting in a large number of rare OTUs. Therefore, OTUs were agglomerated by genera and differential abundance was analyzed between the ileal mucosa for CR and CD calves (Figure 6). These data suggest that feeding colostrum resulted in higher abundance of *Bradyrhizobium* (Proteobacteria), *Clostridium* XIVa and unclassified species of Firmicutes, as well as *Trueperella* (Actinomyces). In contrast, colostrum deprivation resulted in higher levels of colonization with bacteria that dominate within the Firmicutes phyla, and were represented by *Clostridial* species, *Ruminococcus* and *Dorea*, and with other Firmicutes, *Parvimonas* and *Faecalicoccus*. Additionally, CD calves had higher levels of *Pasteurellaceae*, a common intestinal commensal, within the ileal mucosa.

Additional OTUs present within the ileum, regardless of colostrum feeding, included genera such as *Campylobacter*, *Phyllobacteriaceae*, and *Burkholderia*, within phylum Proteobacteria, as well as multiple *Clostridia* species (*sensu stricto* and *Lachnospiraceae*). Overall microbial populations observed in spiral colon and cecum followed similar patterns to the ileum, however, *Actinomyces* ssp. were also observed for these tissues. Abundance between mucosa and lumen contents of the spiral colon and cecum were largely not different due to colostrum feeding, with increased abundance noted only for *Pseudomonas* in the mucosa of both tissues.

## 4. Discussion

To date, this study is the first to explore MAP colonization of intestinal tissues in experimentally infected calves with a terminal endpoint as early as 14 days of age. These results demonstrate that the bacterial pathogen can rapidly colonize in low abundance in distal regions of the small intestine and colon after inoculation with two doses of 10^8^ cfu MAP. In a commercial setting, calves born to infected, “super-shedder” cows are potentially exposed to greater than 10^7^ cfu MAP per gram of maternal feces [30] and may also be exposed to MAP in the colostrum and milk of infected dams [31]. The experimental dose is thus a realistic representation of natural infection and confirms that neonates are at extreme risk of infection within the maternity pen. Observed shedding of MAP in feces at day 7 confirms the risk for horizontal transmission among newborn calves and the level of shedding in the feces is characteristic of calves experimentally inoculated with high doses of MAP [32,33,34]. A study of 18 dairy farms that tested positive for MAP in environmental fecal samples demonstrated that animal age was a factor in fecal transmission [35]. In fact, calves less than three months of age were found to shed MAP in their feces and contributed substantially to the risk of environmental contamination with MAP [35]. Despite the appearance of MAP in the feces of exposed calves, the level of shedding herein and in other studies can be explained by a high level of pass-through shedding, whereby the bacterium is not taken up by the intestine. Although colonization of target tissues was successful, the number of viable MAP recovered per tissue was quite low. Sensitivity of detection may have been improved with the use of liquid culture medium, rather than the standard agar system but was not attempted in the current study [36]. Instead we utilized PCR as a corresponding measure of MAP presence within the tissue and this heightened our sensitivity of detection. The presence of MAP at low levels for such a short infection period was expected, as upon infection it can take many years to progress from a paucibacillary to a multibacillary state. Low-abundance colonization of distal tissues of the small intestine without development of lesions in gut-associated lymphoid tissue noted in the present study agrees with previous reports of the course of experimental MAP infection, which includes uptake by ileal Peyer’s patches, internalization by epithelial lymphocytes, and inhibition of phagolysosome maturation [37]. Indeed, lesions within the tissues are typically seen in cattle in more advanced stages of disease and are associated with a high burden of MAP in the tissue, with few to no lesions noted in young calves and older cows in asymptomatic stage of infection [38,39]. Although the lack of lesions herein would be seemingly intuitive, it was impossible to foresee the impacts that colostrum feeding and shifts in the intestinal microbiome might have on MAP burden at the tissue level.

Supplementation with vitamins A, D_3_, and E did not overtly affect MAP colonization in the gut of calves, however, there was a trend towards higher numbers of infected tissues in calves supplemented with vitamin E (CR-E and CR-ADE). This is an interesting observation as we were expecting that vitamins would improve tissue integrity thereby helping to thwart pathogen invasion. Calves in the present study had more than adequate serum levels of retinol, 25-(OH)-D_3_, and α-tocopherol by day 14, averaging 255 ng/mL, 97 ng/mL, and 2800 µg/mL, respectively [16], suggesting that deficiency of vitamin E was not the causative factor for this trend. Although vitamin E plays significant roles as an anti-oxidant and an immunomodulatory agent [40,41], a comprehensive study of dairy cows in the Netherlands found that feeding 3000 IU/day of vitamin E combined with high plasma levels of vitamin E at dry-off led to an increased incidence of mastitis [42]. Therefore, high levels of supplemental vitamin E for protracted periods may be detrimental to the host, with a suggested role as a pro-oxidant at high doses.

Feeding of colostrum to calves resulted in more frequent recovery of MAP from the intestine of CR calves compared to CD calves, although a low n limited statistical differences. This seems counterintuitive because bovine colostrum contains growth factors such as epidermal growth factor and insulin-like growth factors, as well as antimicrobial factors such as lactoferrin, lactoperoxidase, and cytokines that can function as immunomodulators, thus providing enhanced protection against infection [43,44]. But colostrum also contains TGF-β, which is noted for Th1 suppression [43]. We had previously reported that CD calves exhibited increased CD4+ and CD8+ T-cell populations and lower γδ TCR+ T-cells in the total population of freshly isolated PBMCs compared to calves fed colostrum [45]. Further, Th1 immunity in the neonatal calf may be suppressed by the dominant Th2 immune status of the dam [5]. Indeed, we previously reported that antigen-specific responses by mesenteric lymph node cells were Th2-like for all calves, regardless of colostrum supplementation [45]. These results support that natural immune suppression of the bovine neonate may potentiate increased susceptibility to MAP and this may be compounded by immune regulatory roles of colostrum.

Manipulation of the immune bias in neonatal calves may have broader implications than susceptibility to a sole pathogen such as MAP. With that in mind, we sought to superimpose MAP infection onto the study of the gut microbiome in calves. Nutritional treatments of colostrum and fat-soluble vitamins were hypothesized to enable calves to differentially interact with microbial communities in the mucosa, and that differences between mucosal and luminal communities would coincide with increased health. Vitamin supplementation did not influence the microbiota of calves, suggesting that the fat-soluble vitamins did not have a profound effect on immunological parameters or integrity of the intestinal mucosa during the short-term study period. In contrast, feeding of colostrum did impact the relative abundance of communities between the lumen and mucosa of tissues. When phyla differences were examined, the mucosa was indeed found to harbor increased Proteobacteria, which are generally regarded as opportunists, and decreased Bacteroidetes. This finding was more evident in CD calves compared to CR calves and relates to increased likelihood of clinical scours and depression. We previously noted that CD calves had greater occurrence of these clinical signs [16], conforming to the paradigm for dysbiosis [46]. This correlates with the higher burden of *Campylobacter* observed for CD calves. Colostrum deprived calves also demonstrated a reduced abundance of Firmicutes in the ileal mucosa with suggestions of this pattern in the cecal mucosa as well, that was not observed in CR calves. One suggestion for this is the higher abundance of *Clostridium* (*Lachnospiraceae* and *C. sensu stricto*) noted in the mucosa of CR calves. Other reports have demonstrated that feeding colostrum to neonatal dairy calves improved bacterial colonization of the small intestine, particularly for *Bifidobacterium*, of the phylum Actinobacteria [47]. More recently, it was demonstrated that feeding either fresh or heat-treated colostrum increased *Clostridium* and *Bifidobacterium* and reduced the abundance of *E. coli* in the colon of calves compared to calves that were colostrum deprived [48]. Although the relative abundance of Actinobacteria was lower overall compared to other phyla represented in either the intestinal lumen or mucosa, a pattern of increased colonization was noted in CR calves compared to CD calves. This is a noteworthy observation since MAP is part of this phylum and CR-E and CR-ADE calves had greater involvement of tissues colonized with MAP. This aligned with increased *Bifidobacterium* noted for CR calves compared to CD calves in the spiral colon. In experimentally infected calves, we have observed that the spiral colon is often a site of MAP colonization.

To our knowledge only one prior study has reported microbiome data for calves infected with MAP. Derakhshani et al. [49] were the first to characterize changes in the microbiome of the ileal mucosa and feces of MAP-infected calves over infection periods ranging from two weeks to 12 months. They observed a decreased abundance of Proteobacteria, including *Sphingomonoadaceae* and *Pseudomonadaceae*, and Firmicutes, including *Lactobacillus* and *Epulopiscium*, in the ileal mucosa of MAP-infected calves compared to control noninfected calves. Interestingly, they noted significant correlations between the presence of *Clostridium*, *Turicibacter*, and *Peptostreptococcaceae* with the severity of lesions in the ileal mucosa from MAP-infected calves, suggesting a longitudinal impact of MAP infection on the microbiome of the small intestine. All calves in the present study were inoculated with MAP, enabling us to evaluate specific aspects of nutrition on the colocalization of MAP and other bacterial species in the gut. Results for differential abundance aligned with major genera of *Clostridium* spp. reported by Derakhshani et al. [49] despite the abbreviated 14-day study period and lack of lesions noted in the intestinal tissues. Additionally, the general microbiome also aligned with results observed for rectal swabs from calves collected at 7 days of age [50], suggesting that commonalities shared in the gut microbiome were likely due to normal populations in the gut of young calves.

Others have found that despite similarities between lumen and mucosal communities in regions of the large intestine (cecum and colon), the ileal community structure differed markedly between the lumen contents and mucosa [51]. Further, they determined that the microbiome of the ileal mucosa shared commonality with the mucosa of the large intestine, suggesting that perhaps the mucosal bacteria of the ileum inoculate the large intestine downstream [51]. These findings are similar to the present study with similar microbiome populations observed for the three tissues. The ileal region is a focal region for immune activity with the presence of ileal Peyer’s patches and the ileo-cecal lymph node. Macrophages and dendritic cells present in the lamina propria of the intestine initiate a state of tolerance or hyporesponsiveness to luminal bacteria via regulatory T-cells [52]. Immune regulation of the mucosal microbiome in the ileum would be favorable to the animal’s health as it allows commensal bacteria to populate without induction of a chronic inflammatory state [53]. Microbial richness in feces of pre-weaned calves is known to increase through the first seven weeks of age, with reduced richness observed during diarrheal disturbances [54]. It is possible that an increase in ileal Th1 immunity, as evidenced by higher CD4+ and CD8+ T-cell percentages in CD calves, may limit microbial diversity in the ileal-mucosal inoculum, resulting in increased downstream opportunism in the colonic mucosa [45]. Bacterial phylotypes of the spiral colon lumen in CD calves did correspond with a previous 16S phylogenetic survey of calf feces [54]. The lumen contents were dominated by Firmicutes and Bacteroidetes; with Proteobacteria, Actinobacteria, and Fusobacteria represented as minority phyla. Interestingly, phylum Actinobacteria, which houses both *Mycobacteria* and the beneficial *Bifidobacteria*, was nearly absent from mucosa in CD calves, regardless of tissue. *Bifidobacteria* exhibit preferential growth on milk oligosaccharides in vitro [55,56], but low abundance of the parent phylum suggests that these bacteria are less involved in community structure in pre-weaned calves, in agreement with findings by others [54]. Conversely, the presence of Firmicutes is known to be stable in the intestine and evidence of direct vertical transmission through generations suggests host-specific adaptation of organisms belonging to this phylum, especially of genus *Lactobacillus* [57]. Differential regulation of mucosal communities is an interesting finding in the context of morbidity associated with colostrum deprivation. Little is known about long-term effects of colostrum deprivation on cattle health, particularly concerning susceptibility to chronic infections.

## 5. Conclusions

Results of the present study identify colonization of MAP in the distal jejunum, ileum, and ileo-cecal valve, primary sites of intestinal immune regulation, within 14 days after experimental infection in neonatal calves. Furthermore, calves infected with MAP shed the organism in feces within 7 days after inoculation and thus present risk of horizontal transmission in group-housed settings. Supplementation with vitamins A, D_3_, and E did not influence colonization with MAP or diversity of microbial communities in the intestine but feeding colostrum to calves did have an impact. Colostrum-deprived calves presented with the lowest rate of MAP infection but exhibited dysbiosis in the cecum and spiral colon that was likely related to a lack of passive immunoglobulin and immunomodulatory agents normally present in colostrum. Manipulation of host immunity through nutritive factors warrants exploration as a method of preventing infection in neonates but must also be evaluated as a mediator of greater microbial colonization in key intestinal regions such as the ileum. Although somewhat hindered by the low differential abundance within tissues of calves it is clear that colostrum feeding does have a significant impact on microbial populations in the gut, even within a few weeks of birth. These results suggest that colostrum deprivation could have a significant impact on the ability of calves to establish a balance within the total microbial population. This would lead to dysbiosis and increased morbidity, a finding that is relevant to all species of animals. Feeding good quality and hygienic colostrum should be an essential component of animal husbandry as it can provide critical immune factors to immunologically naive animals until their own immune systems can develop.

## Figures and Tables

**Table 1:** Shedding of *Mycobacterium avium* subsp. *paratuberculosis* in feces of calves on day 7 ^1^.

Treatment	Culture (cfu/g) ^a^
CD	1072 ± 728
CR	521 ± 240
CR-A	1053 ± 691
CF-D	1972 ± 1094
CR-E	1635 ± 890
CR-ADE	153 ± 106

^1^ Treatments included CD: Colostrum-deprived; CR: Colostrum-replete; CR-A: Supplemented with vitamin A; CR-D: Supplemented with vitamin D; CR-E: Supplemented with vitamin E; CR-ADE: Supplemented with vitamins A, D, E; Means ± SEM; *n* = 5 per treatment. ^a^ cfu = colony forming unit.

**Table 2:** Number of calves within treatment group that were culture positive for *Mycobacterium avium* subsp. *paratuberculosis* in select intestinal tissues ^1^.

Treatment	DJ	IL	ICV
CD	0	0	0
CR	1	1	2
CR-A	2	1	2
CF-D	1	1	0
CR-E	1	2	2
CR-ADE	2	3	1

^1^ Treatments included CD: Colostrum-deprived; CR: Colostrum-replete; CR-A: Supplemented with vitamin A; CR-D: Supplemented with vitamin D; CR-E: Supplemented with vitamin E; CR-ADE: Supplemented with vitamins A, D, E; *n* = 5 per treatment group per tissue. Tissues included DJ: Distal jejunum; IL: Ileum; ICV: Ileo-cecal valve.
